# Serial negative response after standard and third (Booster) dose of COVID-19 inactivated vaccine is associated with low vitamin D levels in patients with solid cancers

**DOI:** 10.3389/fmed.2022.898606

**Published:** 2022-07-29

**Authors:** Yifei Ma, Pengfei Zhu, Guanqing Zhong, Dao Wang, Lu Cao, Shenrui Bai, Youlong Wang, Ao Zhang, Xinjia Wang

**Affiliations:** ^1^Department of Orthopedics and Spine Surgery, The Second Affiliated Hospital of Shantou University Medical College, Shantou, China; ^2^Department of Clinical Laboratory, The First Affiliated Hospital of Zhengzhou University, Zhengzhou, China; ^3^Department of Clinical Laboratory, State Key Laboratory of Oncology in South China, Collaborative Innovation Center for Cancer Medicine, Guangdong Key Laboratory of Nasopharyngeal Carcinoma Diagnosis and Therapy, Sun Yat-sen University Cancer Center, Guangzhou, China; ^4^Department of Pediatrics, The First Affiliated Hospital of Zhengzhou University, Zhengzhou, China; ^5^Department of Hematological Oncology, State Key Laboratory of Oncology in South China, Collaborative Innovation Center for Cancer Medicine, Sun Yat-sen University Cancer Center, Guangzhou, China; ^6^Department of General Surgery, Hainan Hospital of PLA General Hospital, Sanya, China; ^7^Department of Orthopedics, Cancer Hospital of Shantou University Medical College, Shantou, China

**Keywords:** third-dose-inactivated COVID-19 vaccine, Vitamin D, serial seronegative response, solid cancer patients, nested case-control study

## Abstract

**Introduction:**

The response is poorly understood to the third dose in patients with cancer who failed the standard dose of inactivated SARS-CoV-2 vaccines (CoronaVac). We aim to assess the immune response to the third dose and identify whether vitamin D deficiency is associated with serial serologic failure in patients with cancer.

**Methods:**

Solid cancer patients (SCP-N) and healthy controls (HCs) who were seronegative after the standard-dose vaccines in our previous study were prospectively recruited, from October 2021 to February 2022, to receive the third dose vaccines and anti-SARS-CoV-2S antibodies were measured. SCP-N who failed the third dose (serial seronegative group, SSG) were matched by propensity scores with the historical standard-dose positive cancer patient group (robust response group, RRG). An exploratory analysis was carried out to validate the role of vitamin D on the serology response.

**Results:**

The multi-center study recruited 97 SCP-N with 279 positive controls as RRG and 82 negative controls as HC group. The seroconversion rate after third-dose vaccination was higher in SCP-N than in HC (70.6% vs. 29.4%, *p* < 0.01). The matched comparison showed that patients in SSG had a significantly lower level of vitamin D and consumption rate than RRG or RRG-B (RRG with third-dose positive) (all *p* < 0.01). None had serious (over grade II) adverse events after the third dose.

**Conclusion:**

Solid cancer patients with second-dose vaccine failure may have a relatively poor humoral response to the third dose of COVID-19 vaccines as compared with the seronegative HC group. The consecutively poor humoral response could be associated with poor vitamin D levels and intake. Vitamin D status and cancer-related immune compromise may jointly affect the humoral response following booster vaccination.

## Highlights

### Question

What is the response rate to the third dose vaccines in patients with solid cancer who failed the standard 2-dose-inactivated (CoronaVac) vaccines?

### Findings

In the prospective standard-dose failure cohort of 97 patients with solid cancers, a response failure rate of 64 of 97 (66%) was observed, and vitamin D deficiency may play a role in serial seronegativity.

### Meaning

The humoral response to the third dose of COVID-19 vaccines was relatively low in seronegative patients with solid cancer, and serial negative may be associated with vitamin D deficiency.

## Introduction

Patients with cancer are susceptible to severe disease course of severe acute respiratory syndrome Coronavirus 2 (SARS-CoV-2, COVID-19) infection due to compromised immune status and immunomodulator treatment (1). Vaccines are the mainstay of preventative measures against the increased risk of death for patients with cancer and they are currently among the most prioritized populations to be vaccinated (2). In China, the vaccination program, orchestrated by the Chinese health administrations, has penetrated each community and the booster dose of inactivated vaccines was recommended for all the inhabitants who finished the last dose 6 months ago (3). However, the debates are still underway on whether satisfactory humoral response could take place for patients with cancer because of altered immune status, and evidence of prior research showed decreased response to the standard dose (1).

In the group of patients with cancer, overall, prior research on mRNA vaccines has demonstrated serologic response comparable to healthy control samples both after the standard dose and after the booster dose of vaccines (4, 5). For inactivated vaccines, our previous retrospective cohort of 302 patients with cancer demonstrated a 69.8% positive response after a standard 2-dose of vaccination with mild and well-managed adverse events (6). As the need for the booster vaccination is desperate in the population with cancer, the question arises as to whether they would be suitable for the third dose in terms of serology and tolerability. A prior report on hematologic malignancies demonstrated that 23% of the enrolled patients had positive responses after the third dose of vaccination, but the data on the solid cancers, especially, in those patients with cancer who failed the standard dose has been scarce (7). Specifically, as nearly 30% of the patients with solid cancer in our previous research failed the standard dose, it is unknown whether the third dose would achieve sufficient humoral response in these patients and what risk factors contribute to the response to the third-dose vaccines.

In addition to the demographic and therapeutic risk variables (aging and chemotherapy status) that may potentially contribute to the seroconversion failure, the nutrition status inherent to the patient’s immune status are to be elucidated (8). Prior research identified vitamin D as a key micro-element to modulate the immune response to COVID-19 infection status, disease course, and prognosis in healthy adults (9). Reviews have postulated vitamin D deficiency as a probable reason for the lack of response to inactivated vaccines in the Asian population, although studies concerning the association between vitamin D and mRNA vaccination outcomes have given inconclusive evidence (10, 11). In the earlier studies, low vitamin D levels were found associated with poor hepatitis B vaccination response (12). In children, vitamin D supplementation was evidenced to be beneficial to influenza vaccine outcomes (13). Given, the high prevalence of vitamin D deficiency in the elderly or female patients with cancer, we hypothesized the response to the inactivated vaccines may be associated with vitamin D status.

Thus, in the current study, we recruited the patients who failed the standard dose to receive booster-dose vaccination to find out what factors are associated with the serologic response, and then a nested case-control comparison was done with the historical cohort to explore whether vitamin D affects the response.

## Materials and methods

### Study design

This prospective, multicenter study recruited solid cancer patients (SCP) who finished standard 2-dose inactivated COVID-19 vaccines (CoronaVac) but had negative serologic status (defined as SCP-N) and two groups of controls to receive the third dose (booster dose) of CoronaVac from October 2021 to February 2022. In the negative control group, the subjects with non-cancer who failed the standard dosage of CoronaVac (defined as healthy control, HC) were recruited to compare the serology difference. In the positive control group, we recruited SCP who tested positive at the standard 2-dose CoronaVac (defined as the robust response group, RRG) to investigate the vitamin D status difference.

All the participants were enrolled from our previous longitudinal Vacan Cohort (COVID-19 Vaccination of Cancer, Shan 2021-137) (6). The inclusion criteria of the current study were as follows: (1) SCP with histological diagnosis of solid cancers irrespective of pathological types; (2) over 18 years old; (3) willing to have the clinical samples reviewed by the study, including blood and feces. Key exclusion criteria were as follows: (1) the prognosis of the SCP was less than 3 months or in the hospice settings; (2) the previous history of severe anaphylaxis to any of the vaccine contents. Ethical approval was obtained at the Second Affiliated Hospital of Shantou University Medical College. Vican study has been performed according to the ethical principles of the Declaration of Helsinki, Good Clinical Practice, and all the patients provided informed consent before participation.

The primary outcome of the study was the comparison of the seroconversion rate between SCP-N and HC after the third dose (booster dose) of CoronaVac. In SCP who were negative both at the standard dose and booster CoronaVac (defined as a serial seronegative group, SSG), an exploratory analysis was carried out to compare the vitamin D status both with RRG and RRG with a positive response after the booster dose of CoronaVac (RRG-B). The secondary goal was to report the follow-up adverse events following vaccination.

### Baseline and follow-up data

Demographic and therapeutic data were extracted from the Case Record Form of the five tertiary referral hospitals of the Vacan cohort, and electronic data capture systems were applied to save and monitor the de-identified profiles. The demographic data included age, sex, pathology types, rheumatic comorbidity, and patient performance scores (ECOG-PSs). Treatment information included immune checkpoint inhibitors (ICIs, or PD-1 blockers), endocrine treatment for breast cancers, and chemotherapy status. The status was quantified by the time elapsed from the last chemotherapy.

Patients recruited into the study received the third dose at the registered vaccination site of the Center for Disease Control and Prevention, and whole blood was collected in EDTA tubes and stored at −80°C until processing. The time of blood draw and lab processing that elapsed from the vaccination time was recorded, and detailed methods of serology test have been described previously (6).

After all the results of serum COVID-19 antibody titers were completed, the level of vitamin D was tested in SSG and RRG. Vitamin D status was evaluated and represented by serum 25-hydroxyvitamin D (25(OH)D) concentrations. Measurements were done with a chemiluminescent immunoassay with the Cobas-E automated systems along with the Elecsys 25-VitDs kit. The measurement range of the assay is from 4 to 110 ng/ml, and readings below 20 ng/ml (50 nmol/L) were defined as vitamin D deficiency. The patients were also followed-up on supplement consumption for the past 6 months.

### Statistics and analytic protocols

The comparison of the seroconversion rate between SCP-N and HC after the third dose (booster dose) of CoronaVac was conducted. To alleviate potential bias between the two comparison groups, participants were matched by propensity scores to reach head-to-head comparison as further validation. The propensity score-matched (PSM) analysis was carried out in a 1:1 manner with a multivariate conditional logistic regression model with a caliper width of 0.1 (14). Finally, the univariate and multivariate regression analyses were used to find out the risk factors of third-dose vaccine failure in the high-risk population (SCP-N), then, a user-friendly nomogram that incorporated the pre-test variables was illustrated.

The nomogram was formulated in the SCP-N by using the package of ‘‘rms’’ and ‘‘foreign’’ in R version 4.0.5^1^. The performance of the nomogram was evaluated by concordance index (C-index) and by comparing nomogram-predicted vs. observed rates of seroconversion failure. Bootstraps with 1,000 resample were used for these activities. During the internal validation of the nomogram, the total points of each patient in the cohort were calculated according to the established nomogram.

A nested case-control study design was applied to compare the vitamin D status between SSG and RRG. To further decrease potential bias across the geographical and chronological difference in patient enrollment, patients in the SSG and RRG were also matched by propensity scores. The comparison of SSG with RRG-B was conducted similarly. The model applied in the PSM involved the random, 1:2 nearest-neighbor method with a caliper width of 0.1 (14).

The variables to enter the regression model of all the PSM analyses included the baseline, therapeutic, and time-related data of each patient. To evaluate the matching performance, the standardized mean difference (SMD) was calculated in each matched variable according to Austin PC et al., and any SMD above (√ ((n1 + n2)/n1*n2)*1.96 would be considered as an imbalanced matching variable (n1 and n2 represent the sizes of the matched 2 variables) (14). The statistics applied in propensity score matching were carried out in the SPSS V.23.0 software.

The categorical variables were represented as numbers and percentages, and the continuous variables were illustrated as means ± standard deviations (SDs). The power (1-β) of the statistical test was calculated according to the sample size and was performed on the PASS software (V.15.0), and each test (excluding *post hoc* analysis) was based upon a pre-specified statistical hypothesis with a type I error of 0.05 (15). As the statistical test of the hypothesized difference was *post hoc* analysis, the type I error was set at 0.025 to prevent data fishing. Statistic tests involved the χ Square test or Fisher exact test in the categorical variables, the independent Student *t*-test in continuous variables, and paired *t*-test and McNemar’s test in the matched samples.

## Results

### Participant characteristics

The study identified 121 SCP-N, 88 HC, and 279 RRG participants. In total, 24 SCP-N and 6 HC participants did not receive the third dose of the vaccine because of insufficient time since the last dose (less than 6 months) or rejection to vaccinate. Thus, 376 patients with cancer (including 97 patients in the SCP-N group and 279 patients in the RRG) and 82 HC participants were included. The SCP-N group included 19 (19.6%) men and 78 (80.4%) women, with a mean age of 48.9 ± 12.0 years, as compared with 21 (25.6%) men and 61 (74.3%) women in the HC group. No patients were infected with the COVID-19 virus or developed COVID-19-related diseases during the study period, the pathology types of SCP-N fell into four categories: gastrointestinal epithelial cancers (GI, 31 patients, 32.0%), head and neck cancers (HN, 23 patients, 23.7%), non-small cell lung cancers (NSCLC, 12 patients, 12.3%), and breast cancers (BC, 31 patients, 32.0%). In total, 35 (36.1%) patients received chemotherapy within the past 3 weeks, and the mean time elapsed from the last cycle of all the patients was 5.60 ± 3.84 weeks. The rheumatic comorbidity was found in 27 (27.8%) SCP-N and 7 (8.5%) HC. In total, nine (9.2%) SCP-N were in the metastatic stage of cancer. The baseline characteristics of the SCP-N and HCs were shown in Supplementary Table 1.

### Response rates in solid cancer and healthy control groups

After the third dose of vaccination, 64 (66.0%) SCP-N had a negative serologic response and were thus defined as the patients in SSG, and 33 (34.0%) had a positive serologic response. The mean serologic titer of SCP-N was 150.4 ± 300.3 U/ml. In the HC group, the seroconversion rate of third-dose vaccination was 29.2%, with the mean serologic titer of 1,023.44 ± 984.4 U/ml.

**FIGURE 1:**
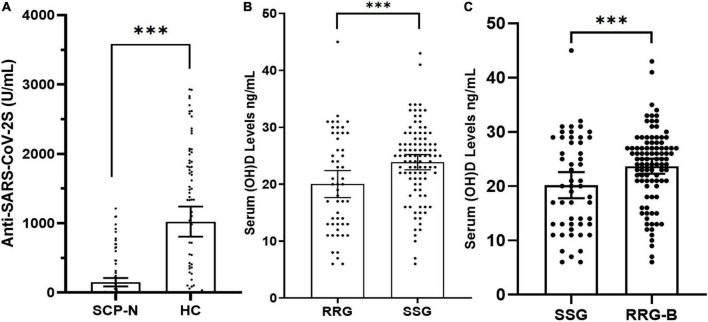
**(A)** Dot plot of anti-SARS CoV-2S serology titers after the third dose of COVID-19 vaccines in solid cancer patients (SCP) and healthy control (HC) participants with standard-dose vaccine negativity. **(B)** Matched dot plot of vitamin D levels in the serial seronegative group (SSG) and the robust response group (RRG). **(C)** Matched dot plot of vitamin D levels in the serial seronegative group (SSG) and the robust response group-booster (RRG-B). The symbol “***” denotes “<0.001”.

The SCP-N were matched with HC with variables of age and sex entering the PSM regression calculation. The matching yielded 68 pairs, with details shown in Table 1. After the booster vaccination, the cohort of SCP-N still comprised a significantly larger part of patients who were tested seronegative (*n* = 48, 70.6%) than the healthy control cohort (*n* = 20, 29.4%, *p* < 0.01, see Table 1). The serologic titers of HC were significantly higher than those of the SCP-N (112.18 ± 243.6 vs. 1,043.36 ± 993.1 U/ml, *p* < 0.01, Figure 1A).

**TABLE 1:** Propensity score-matched comparison between cancer patients and healthy control after failed standard-dose vaccination.

Factor		SCP-N	HC	*p*
**Matched Baseline Variables**				
Age (years)		49.00 (11.00)	48.72 (10.94)	0.88
Sex	Male	14 (20.6)	14 (20.6)	1.00
	Female	54 (79.4)	54 (79.4)	
**Follow-up Variables**				
Serology Response	Positive	20 (29.4)	48 (70.6)	<0.01
	Negative	48 (70.6)	20 (29.4)	
Serology Titers, U/mL		112.18 (243.61)	1043.36 (993.14)	<0.01

SCP-N, solid cancer patients who were seronegative after standard-dose vaccination; HC, healthy control.

**FIGURE 2:**
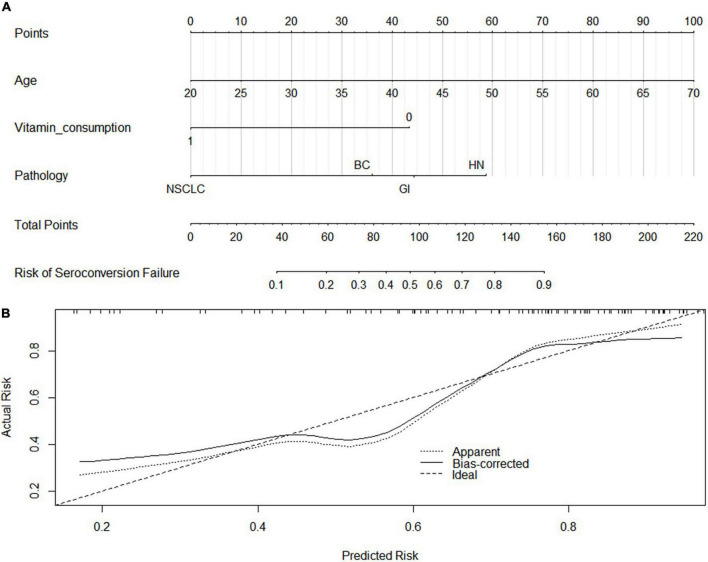
Nomogram Development **(A)** by using the significant variables in the regression analysis and calibration curve **(B)**. NSCLC, Non-small cell lung cancer; GI, gastrointestinal cancers; HN, head and neck cancers; BC, Breast Cancers. The C-index and calibration curve was derived based on the regression analysis. The C-index was 0.8 and the internal calibration curve was shown.

Univariate logistic regression analysis of serology response rate of booster vaccination in SCP-N showed that seronegative was associated with the following variables (*p* < 0.1, Table 2) which were put into the multivariate regression model: age, sex, pathology types, time to last chemotherapy, ECOG-PS, vitamin D supplement consumption, and vitamin D levels. Multivariate regression identified the following variables to be significant: age (OR = 1.14, 95% CI = 1.05–1.23, *p* < 0.01), pathology types (OR = 17.72 for NSCLC and 31.37 for HN cancers with reference of BC, respectively), time to last chemotherapy (OR = 0.84, 95% CI 0.71–0.10, *p* = 0.045), vitamin D supplement consumption (OR = 0.24, 95% CI 0.07–0.80, *p* = 0.02), and vitamin D levels (*p* = 0.02, OR = 0.92, 95% CI = 0.86–0.98). A nomogram was thus built with pre-serologic variables to predict seroconversion failure in SCP-N (Figure 2).

**TABLE 2:** Univariate and multivariate regression analysis for serology response rate of booster vaccination in SCP-N.

Variables		Serology response		*p* (Univariate)	*p* (Multivariate)	Odds Ratio	95% CI
					
		Positive (*N* = 33, 34%)	Negative (*N* = 64, 66%)				
Age (years)	≤ 50	23 (69.7)	34 (53.1)	<0.01	<0.01	1.14	1.05 – 1.23
	> 50	10 (30.3)	30 (46.8)				
	Mean (SD)	44.03 ± 13.18	51.34 ± 13.54				
Sex	Male	6 (18.2)	13 (20.4)	0.06	0.96	1.04	0.21 – 5.23
	Female	27 (81.8)	51 (79.6)		Reference	Reference	Reference
Pathology	NSCLC	6 (18.1)	6 (9.3)	0.04	0.02	17.72	1.57 – 199.45
	GI	8 (24.2)	23 (35.9)		0.06	7.14	0.96 – 53.23
	HN	4 (12.1)	19 (29.6)		0.00	31.37	3.03- 324.67
	BC	15 (45.4)	16 (25.2)		Reference	Reference	Reference
Time to Last Chemotherapy (weeks)	≤ 3	12 (36.3)	23 (36.0)	0.09	0.05	0.84	0.71 – 0.10
	> 3	21 (63.6)	41 (64.0)				
	Mean (SD)	6.52 ± 4.55	5.13 ± 3.35				
ECOG-PS	0	14 (42.4)	42 (65.6)	0.03	0.49	0.64	0.18 – 2.26
	1	19 (57.6)	22 (34.4)		Reference	Reference	Reference
Comorbidity with Rheumatic Disease	Yes	12 (36.4)	15 (23.5)	0.18	—		
	No	21 (63.6)	49 (76.5)				
PD-1B treatment	Yes	7 (21.2)	12 (18.7)	0.77	—		
	No	26 (78.7)	52 (82.3)				
Time between 3^rd^ and 2^nd^ dosage	6 months	18 (54.5)	34 (53.1)	0.89	—		
	7 months	15 (45.5)	30 (46.9)				
Test time after the third dose	2-3 weeks	9 (27.3)	17 (26.6)	0.69	—		
	3-4 weeks	15 (45.5)	21 (32.8)				
	4-5 weeks	9 (27.2)	26 (40.6)				
Metastatic Status	Yes	4 (12.1)	5 (7.8)	0.49	—		
	No	29 (87.8)	59 (92.2)				
Vitamin Supplement Consumption^†^	+	23 (69.7)	27 (42.2)	0.01	0.02	0.24	0.07 – 0.80
	–	10 (30.3)	37 (57.8)		Reference	Reference	Reference
Vitamin D Levels (ng/mL)		28.9 ± 11.1	22.0 ± 9.1	< 0.01	0.02	0.92	0.86 – 0.98
Endocrine Treatment	Yes	9 (27.3)	9 (14.1)	0.12	—		
	No	24 (72.7)	55 (85.9)				

SCP-N, solid cancer patients who were seronegative after standard-dose vaccination; 95%CI, 95% confidence interval; SD, standard deviation; NSCLC, Non-small cell lung cancer; GI, gastrointestinal cancers; HN, head and neck cancers; BC, Breast Cancers; ECOG-PS, Eastern Cooperative Oncology Group-Performance Score; PD-1B, PD-1 blockers; ^†^, Daily consumption of vitamins (including D) at least five days per week during the past 6 months.

### Vitamin D status in the serial seronegative group and the robust response group

The SSG data after the third vaccination were matched with the RRG data to compare the hypothesized difference in vitamin D status. The demographic and therapeutic variables to be matched included the following: age, sex, pathology types, time to last chemotherapy, ECOG-PS, rheumatic comorbidity, PD-1B treatment, serologic test time, endocrine treatment, and metastasis status.

Before matching, there were 279 patients in the RRG and 64 patients in the SSG, and after matching there were 53 patients in the SSG which matched with 98 patients in the RRG. The detailed balance tests of each variable were shown in Supplementary Table 2 and Supplementary Figure 1. According to the formula (√ ((n1 + n2)/n1*n2) *1.96, the largest imbalance limit was 0.33, and the SMD of each variable after PSM was less than 0.33.

Before matching, the vitamin D levels in SSG and RRG were 22.0 ± 9.1 ng/ml and 23.14 ± 6.26 ng/ml, respectively, and there was no significant difference in Vitamin D levels between the two groups (*p* = 0.24). In total, 26 (40.6%) patients in SSG and 57 (20.4%) patients in RRG had vitamin D deficiency, respectively, and there was a significant difference between the two groups (*p* < 0.01). In total, 27 (42.2%) patients in SSG and 155 (55.6%) patients in RRG reported at least 5 days of vitamin D-containing supplement consumption over one week during the past 6 months, respectively, and the difference reached the borderline significance (*p* = 0.05). After matching, the vitamin D levels were 20.0 ± 8.6 ng/ml in SSG and 23.9 ± 6.7 ng/ml in the RRG (*p* < 0.01, Figure 1B). Also, 26 (49.1%) patients in SSG and 22 (22.4%) patients in RRG had vitamin D deficiency, respectively, with a significantly higher rate of deficiency in the SSG (*p* < 0.01, Table 3). In total, 17 (32.1%) patients in SSG and 76 (77.6%) patients in RRG reported vitamin D consumption, and the RRG had a significantly higher rate than the SSG (*p* < 0.01).

**TABLE 3:** Propensity score-matched comparison between serial SSG and RRG.

Factor		SSG	RRG	*p*
**Matched Baseline Variables**				
Age (years)		49.6 (10.4)	50.2 (11.1)	0.77
Gender	Male	10 (18.9)	26 (26.5)	0.29
	Female	43 (81.1)	72 (73.5)	
Pathology	NSCLC	6 (11.3)	14 (14.3)	0.73
	GI	20 (37.7)	43 (43.9)	
	HN	11 (20.8)	15 (15.3)	
	BC	16 (30.2)	26 (26.5)	
Time to Last Chemotherapy (weeks)		4.3 (2.8)	3.6 (3.7)	0.28
ECOG-PS	0	36 (67.9)	70 (71.4)	0.65
	1	17 (32.1)	28 (28.6)	
Metastasis	Yes	4 (7.5)	8 (8.2)	0.90
	No	49 (92.5)	90 (91.8)	
PD-1B Treatment	Yes	11 (20.8)	17 (17.3)	0.61
	No	42 (79.2)	81 (82.7)	
Endocrine Treatment	Yes	9 (17.0)	17 (17.3)	0.96
	No	44 (83.0)	81 (82.7)	
Serology Test Time (weeks)	1-2	16 (30.2)	29 (29.6)	0.92
	3-4	16 (30.2)	27 (27.6)	
	5-6	21 (39.6)	42 (42.8)	
Comorbidity with Rheumatic Disease	Yes	12 (22.6)	22 (77.6)	0.99
	No	41 (77.4)	76 (22.4)	
**Follow-up Variables**				
Vitamin Supplement Consumption	+	17 (32.1)	76 (77.6)	< 0.01
	–	36 (67.9)	22 (22.4)	
Vitamin D Levels (ng/mL)		20.0 (8.6)	23.9 (6.7)	< 0.01
Vitamin D	Yes	26 (49.1)	22 (22.4)	< 0.01
Deficiency	No	27 (50.9)	76 (77.6)	

SSG, serial seronegative group, defined as cancer patients who had negative serology after both standard-dose and booster-dose COVID-19 vaccination; RRG, robust response group, defined as cancer patients who had positive serology after the standard-dose COVID-19 vaccination; PD-1B, PD-1 blockers; NSCLC, Non-small cell lung cancer; GI, gastrointestinal cancers; HN, head and neck cancers; BC, Breast Cancers; ECOG-PS, Eastern Cooperative Oncology Group-Performance Score.

Of the 279 patients in RRG, 251 patients who finished the booster vaccine 6 months after the standard vaccination were successfully followed up with vitamin D status and serology results after the booster vaccination. There were 230 patients (91.6%) who were tested seropositive and were thus included in the second positive control cohort (RRG-B). The PSM analysis was performed between SSG and RRG-B that included the following variables, namely, age, sex, pathology types, time to last chemotherapy, ECOG-PS, rheumatic comorbidity, PD-1B treatment, serologic test time, endocrine treatment, and metastasis status. After matching, a total of 52 patients in the SSG were matched with 97 patients in the RRG-B (Table 4). The vitamin D levels were 20.2 ± 8.7 ng/ml in SSG and 23.7 ± 6.9 ng/ml in the RRG-B (*p* < 0.01, Figure 1C). The vitamin D supplement consumption and vitamin D deficiency status were significantly different between the two groups (*p* < 0.01).

**TABLE 4:** Propensity score-matched comparison between SSG and RRG-B.

Factor		SSG	RRG-B	*p*
**Matched Baseline Variables**				
Age (years)		49.94 (10.61)	50.80 (10.05)	0.63
Gender	Male	10 (19.2)	16 (19.5)	0.68
	Female	42 (80.8)	81 (83.5)	
Pathology	NSCLC	6 (11.5)	13 (13.4)	0.96
	GI	20 (38.5)	40 (41.2)	
	HN	11 (21.2)	19 (19.6)	
	BC	15 (28.8)	25 (25.8)	
Time to Last Chemotherapy (weeks)		4.10 (2.63)	4.01 (3.53)	0.88
ECOG-PS	0	34 (65.4)	60 (61.9)	0.67
	1	18 (34.6)	37 (38.1)	
Metastasis	Yes	4 (7.7)	9 (9.3)	0.74
	No	48 (92.3)	88 (90.7)	
PD-1B Treatment	Yes	11 (21.2)	23 (23.7)	0.72
	No	41 (78.8)	74 (76.3)	
Endocrine Treatment	Yes	8 (15.4)	16 (16.5)	0.86
	No	44 (84.6)	81 (83.5)	
Serology Test Time (weeks)	1-2	16 (30.8)	26 (26.8)	0.86
	3-4	15 (28.8)	31 (32.0)	
	5-6	21 (40.4)	40 (41.2)	
Comorbidity with Rheumatic Disease	Yes	14 (26.9)	21 (21.6)	0.47
	No	38 (73.1)	76 (78.4)	
**Follow-up Variables**				
Vitamin D Supplement Consumption	+	18 (34.6)	58 (59.8)	< 0.01
	–	34 (65.4)	39 (40.2)	
Vitamin D Levels (ng/mL)		20.19 (8.68)	23.67 (6.89)	< 0.01
Vitamin D	Yes	25 (48.1)	21 (21.6)	< 0.01
Deficiency	No	27 (51.9)	76 (78.4)	

SSG, serial seronegative group, defined as cancer patients who had negative serology after both standard-dose and booster-dose COVID-19 vaccination; RRG-B, robust response group-booster, defined as cancer patients who had positive serology after both standard-dose and booster-dose COVID-19 vaccination; PD-1B, PD-1 blockers; NSCLC, Non-small cell lung cancer; GI, gastrointestinal cancers; HN, head and neck cancers; BC, Breast Cancers; ECOG-PS, Eastern Cooperative Oncology Group-Performance Score.

### Adverse events

**FIGURE 3:**
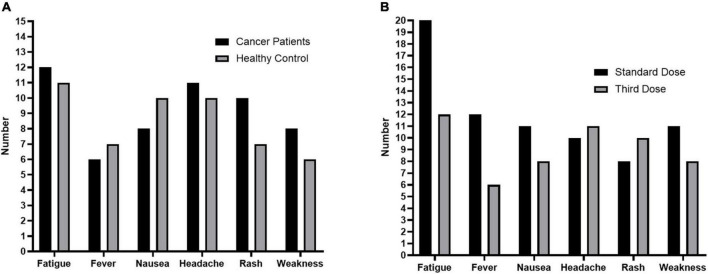
Adverse Events in the study. **(A)**, Adverse events after the third dose in solid cancer patients who were seronegative after standard-dose vaccination (SCP-N, *n* = 97) and healthy control participants (HC, *n* = 82). **(B)**, Adverse events after the third dose and standard dose of COVID-19 vaccination in SCP-N.

After the third dose of vaccination, 33 (34.0%) SCP-N and 25 (30.4%) HC participants reported at least one systemic reaction, but all the reactions were mild and self-limited. The most frequently reported reactions in patients with cancer were fatigue (12.3%), fever (6.1%), nausea (8.1%), headache (11.3%), rash (10.3%), and weakness (8.2%, Figure 3).

## Discussion

The current report was an extended study recruiting for vaccination for patients with cancer (Vacan cohort) who failed the standard 2-dose inactivated vaccines. Overall, the inactivated vaccines were tolerated as much in this study as in the previous report, although the seroconversion failure rate (66.0%) of the third dose of vaccination was much higher than the standard dose (30.1%) (6). Then, the exploratory analysis identified that vitamin D status was associated with the serology by the means of nested case-control comparisons. At last, a user-friendly nomogram that incorporated the pre-test variables was illustrated to help the clinicians find high-risk patients who may fail the third dose of vaccination. To date, this was the first report on serology outcomes for seroconversion failure patients with solid cancers after inactivated vaccination (CoronaVac).

Among the matched participants who were seronegative after the standard dose of vaccination, the third-dose seroconversion failure rate of SCP-N was shown significantly higher than the HC (70.6 vs. 29.4%), and the serologic titers were also lower. In line with our previous study that the decreased seroconversion rate was seen in patients with cancer compared with the healthy controls after receiving the standard dose of inactivated COVID-19 vaccines, the current results of lower third dose seroconversion rate in SCP-N may be associated with compromised immune status (6). However, the seronegative rate of the third dose vaccine among SCP-N was doubled compared with the standard-dose seronegative rate among patients with cancer, which indicated probable interaction of the compromised immune status with other risk factors of the body. Although the poor performance of seroconversion status of third dose vaccination among SCP-N was observed, SCP-N may have a higher booster-dose seroconversion rate (34.0% in 97 patients) than hematologic malignancies (23.8% of 172 patients) as Herishanu et al. reported (7). Similarly, patients in both the studies received vaccines at the 6 to 7 months post the second dose, when most cancer patients’ vaccine serological levels declined (7).

In this study, we identified several independent risk factors associated with seroconversion failure, namely, age, pathology types, and the time elapsed from the last chemotherapy. Aging was significantly associated with poor-serology response, and this may be because of the compromised immune systems in the elderly population. Pathology types and chemotherapy status also impact the immune function, though PD-1B treatment was found to be not associated with serology outcome. These risk factors were similar to the variables found in the regression analysis after the standard vaccines in the prior report (6). However, multivariable regression analysis also identified vitamin D status as an independent risk factor, namely, vitamin D levels and vitamin D supplement consumption. This may be considered as the evidence of the interaction of compromised immune status with vitamin D in patients with cancer.

To further validate the role of vitamin D in affecting the serology status of inactivated COVID-19 vaccination among SCP, we compared the vitamin D status of SSG with RRG, which may be considered a comparison of two extremes. The results showed that even after controlling for other significant variables through PSM analysis, the vitamin D levels and supplement consumption of SSG were still significantly lower than the RRG, suggesting that poor vitamin D levels and consumption might play a role in the weakened immune response.

Up to date, the role of vitamin D in moderating the response to COVID-19 vaccines is still controversial. In the Asian population, these were generally decreased titers of serology as compared with the white population, and one study postulated that low vitamin D status may play a role (6, 9). However, one previous research on the vitamin D status of subjects with non-cancer found no significant association between vitamin D status and serology response in a German sample of 126 participants (11). The moderating effect probably relies on its influence on the adaptive immune response. It was reported that vitamin D may improve CD4 + lymphocyte production, inhibit T helper cell proliferation, and thus, promote the production of virus-specific antibodies by activating B cells into plasma cells (16). As the sample size of this SSG was relatively small, more prospective studies are encouraged to give a definitive conclusion.

This work bears several limitations. Although the patients in SSG were prospectively enrolled and PSM analysis was carried out to balance confounding bias, it should be noted that the patients in RRG were in the retrospective cohorts, and thus, the comparison results should be interpreted with caution. Second, the sample size of the SSG cohort was relatively small, and the result should be validated with larger sample sizes. In addition, the demographic composition was not homogeneous, with a much higher proportion of female participants, and thus, the results should be interpreted cautiously. It should also be noted that the poor response in the SCP–N was due to the immunosuppression state linked to the neoplasm, which has been supported in prior studies. (1) The study did not separately illustrate the role of vitamin D in moderating serologic response in the general population, which encourages future investigation.

## Conclusion

Patients with solid cancer who failed the standard 2-dose inactivated COVID-19 vaccines may have a relatively poor humoral response to the third dose of vaccines as compared with the seronegative healthy controls. As compared with the standard-dose seroconversion, the consecutively poor-humoral response could be associated independently with the poor vitamin D levels and intake. Vitamin D status and cancer-related immune compromise may jointly affect the humoral response following the booster vaccination.

## Data availability statement

The raw data supporting the conclusions of this article will be made available by the corresponding author upon reasonable request.

## Ethics statement

The studies involving human participants were reviewed and approved by The Second Affiliated Hospital of Shantou University Medical College. The patients/participants provided their written informed consent to participate in this study. Written informed consent was obtained from the individual(s) for the publication of any potentially identifiable images or data included in this article.

## Author contributions

XW and AZ conceptualized and designed the study and reviewed and revised the manuscript. YM and PZ designed the data collection instruments, carried out the initial analyses, and wrote the first draft of the manuscript. GZ coordinated and supervised data collection and critically reviewed the manuscript for important intellectual content. DW, LC, SB, and YW collected data and reviewed and revised the manuscript. All authors approved the final manuscript as submitted and agreed to be accountable for all aspects of the work.

## Footnotes

1
http://www.r-project.org/
