# Supplementary material for: Serial negative response after standard and third (Booster) dose of COVID-19 inactivated vaccine is associated with low vitamin D levels in patients with solid cancers
Source: Front Med (Lausanne). 2022 Jul 29;9:898606. doi: 10.3389/fmed.2022.898606 (PMC9373038; doi:10.3389/fmed.2022.898606)
Supplement: Supplementary file 1 [file Data_Sheet_1.docx]

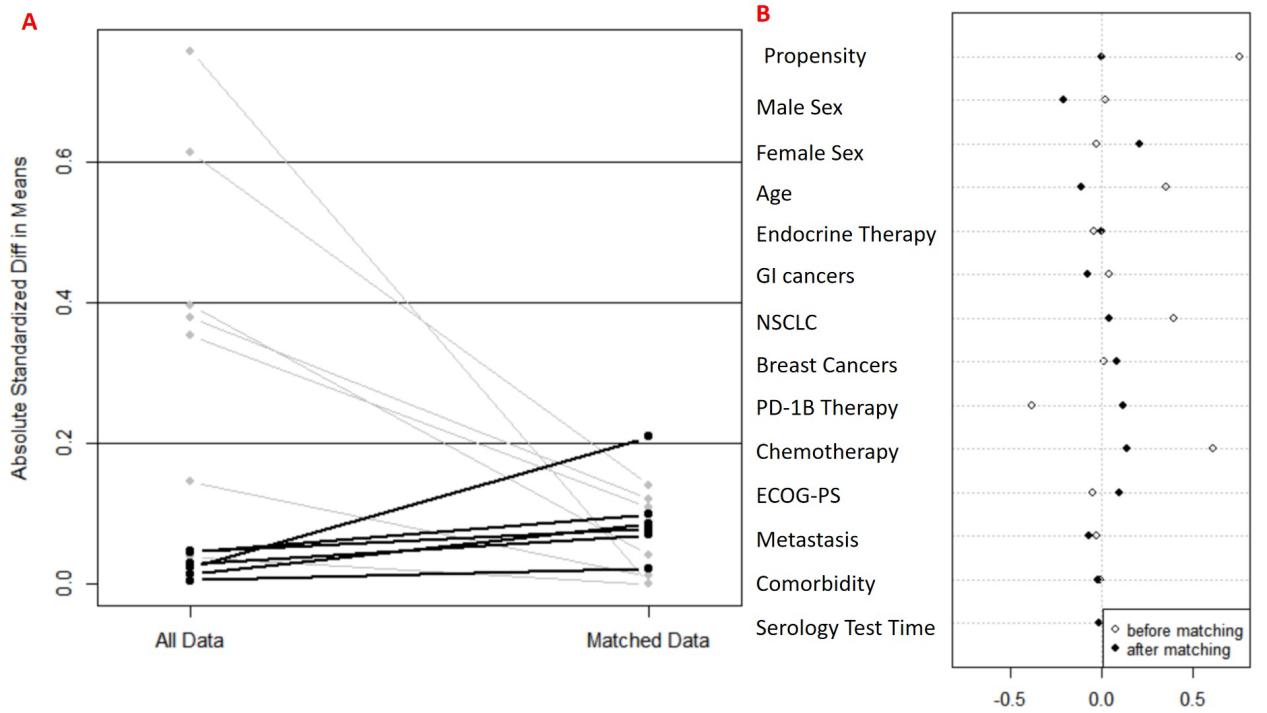


**Supplementary Figure 1.** Efficacy evaluation of propensity score matching between serial seronegative group (SSG, defined as cancer patients who had negative serology after both standard-dose and booster-dose COVID-19 vaccination) and robust response group (RRG, defined as cancer patients who had positive serology after the standard-dose COVID-19 vaccination). 2A, standardized mean differences (SMD) in unmatched and matched samples; 2B, dot plot of SMD of each variable that entered the matching.

| **Supplementary Table 1. Baseline Variables of Seronegative Participants after the Standard-dose COVID-19 Vaccination** | | | |
| --- | --- | --- | --- |
| **Variables** | | **SCP-N** | **HC** |
| Age (years) | ≤ 50 | 53 (54.6) | 39 (47.5) |
|  | > 50 | 44 (45.3) | 43 (52.4) |
|  | Mean (SD) | 48.9 (12.0) | 48.6 (10.8) |
| Sex | Male | 19 (19.6) | 21 (25.6) |
|  | Female | 78 (80.4) | 61 (74.3) |
| Pathology | NSCLC | 12 (12.3) | — |
|  | GI | 31 (32.0) | — |
|  | HN | 23 (23.7) | — |
|  | BC | 31 (32.0) | — |
| ECOG-PS | 0 | 56 (57.8) | — |
|  | 1 | 41 (42.2) | — |
| Comorbidity with Rheumatic Disease | Yes | 27 (27.8) | 7 (8.5) |
|  | No | 70 (72.2) | 75 (91.4) |
| Metastatic Status | Yes | 9 (9.2) | — |
|  | No | 88 (90.7) | — |
| SCP-N, solid cancer patients who were seronegative after standard-dose vaccination; HC, healthy control; SD, standard deviation; NSCLC, Non-small cell lung cancer; GI, gastrointestinal cancers; HN, head and neck cancers; BC, Breast Cancers; ECOG-PS, Eastern Cooperative Oncology Group-Performance Score. | | | |

| **Supplementary Table 2. Imbalance Test results between matched variables in SSG and RRG** | | | | | | | | |
| --- | --- | --- | --- | --- | --- | --- | --- | --- |
| Covariates | SSG | | RRG | | Standard Difference of RRG | | Standardized Mean Difference | |
|  | Before | After | Before | After | Before | After | Before | After |
| Propensity | 0.31 | 0.24 | 0.16 | 0.24 | 0.12 | 0.14 | 0.76 | 0 |
| Male | 0.2 | 0.19 | 0.19 | 0.27 | 0.4 | 0.45 | 0.02 | -0.21 |
| Female | 0.8 | 0.81 | 0.81 | 0.73 | 0.4 | 0.45 | -0.02 | 0.21 |
| Age | 51.34 | 49.64 | 47.61 | 50.78 | 10.98 | 11.16 | 0.35 | -0.11 |
| Endocrine Treatment | 0.14 | 0.17 | 0.15 | 0.17 | 0.36 | 0.38 | -0.04 | 0 |
| GI | 0.36 | 0.38 | 0.34 | 0.42 | 0.47 | 0.5 | 0.05 | -0.08 |
| HN | 0.3 | 0.21 | 0.11 | 0.19 | 0.32 | 0.39 | 0.4 | 0.04 |
| BC | 0.25 | 0.3 | 0.24 | 0.26 | 0.43 | 0.44 | 0.01 | 0.09 |
| PD-1B | 0.19 | 0.21 | 0.34 | 0.16 | 0.47 | 0.37 | -0.38 | 0.12 |
| Time to last chemotherapy | 5.13 | 4.26 | 3.06 | 3.79 | 3.28 | 3.81 | 0.61 | 0.14 |
| ECOG-PS | 0.34 | 0.32 | 0.37 | 0.27 | 0.48 | 0.45 | -0.05 | 0.1 |
| Metastasis | 0.08 | 0.08 | 0.09 | 0.09 | 0.28 | 0.29 | -0.03 | -0.07 |
| Comorbidity | 0.23 | 0.23 | 0.24 | 0.24 | 0.43 | 0.43 | -0.01 | -0.02 |
| Serology Test Time | 2.14 | 2.09 | 2.02 | 2.1 | 0.82 | 0.85 | 0.15 | -0.01 |
| SSG, serial seronegative group, defined as cancer patients who had negative serology after both standard-dose and booster-dose COVID-19 vaccination; RRG, robust response group, defined as cancer patients who had positive serology after the standard-dose COVID-19 vaccination; PD-1B, PD-1 blockers; GI, gastrointestinal cancers; HN, head and neck cancers; BC, Breast Cancers; ECOG-PS, Eastern Cooperative Oncology Group-Performance Score. | | | | | | | | |
